# Commentary: Peptide-Based Targeting of the L-Type Calcium Channel Corrects the Loss-of-Function Phenotype of Two Novel Mutations of the *CACNA1* Gene Associated With Brugada Syndrome

**DOI:** 10.3389/fphys.2021.682567

**Published:** 2021-06-09

**Authors:** Michelle M. Monasky, Carola Rutigliani, Emanuele Micaglio, Carlo Pappone

**Affiliations:** ^1^Arrhythmology Department, IRCCS Policlinico San Donato, San Donato Milanese, Milan, Italy; ^2^Vita-Salute San Raffaele University, Milan, Italy

**Keywords:** Brugada syndrome, L-type calcium channel, CACNA1C, mutations, drug, pharmaceutical, personalized medicine

We read with great interest a recently published article by Di Mauro et al. (2020) describing for the first time the use of a mimetic peptide (R7W-MP) to restore impaired forward trafficking and reduced half-life of L-type calcium channels (LTCC) caused by mutations in the *CACNA1C* gene, restoring channel function *in vitro*. The two novel mutations in the *CACNA1C* gene (Cavα1.2 T320M and Cavα1.2 Q428E) were found in patients with Brugada syndrome (BrS), one asymptomatic (T320M), and one with a history of cardiac arrest, ICD placement, two episodes of self-terminating polymorphic ventricular tachycardia, and runs of atrial fibrillation (Q428E). The mutations in the *CACNA1C* gene, encoding for the pore-forming unit (Cavα1.2), studied in HEK293 cells, exhibited reduced protein trafficking to, and half-life in, the membrane, resulting in reduced calcium current.

Variants in more than 26 different genes have been implicated in BrS (Monasky et al., 2020), the most accepted being the *SCN5A* gene, encoding for the sodium voltage-gated channel alpha subunit 5, or the Na_V_1.5 protein. While some studies have suggested a role for the *CACNA1C* gene in BrS (Fukuyama et al., 2014), the causative effect of the *CACNA1C* gene in BrS has been recently challenged (Hosseini et al., 2018; London, 2019; Wilde et al., 2019), citing the lack of systematic, evidence-based evaluations supporting the causality of this gene. Thus, systematic, evidence-based evaluations are of utmost importance, after several studies suggested an important role for calcium in BrS (Antzelevitch et al., 2007; Cordeiro et al., 2009; Burashnikov et al., 2010; Hoogendijk et al., 2011; Betzenhauser et al., 2015; Monasky et al., 2018).

Calcium plays a pivotal role in cardiac contractility, and the control of intracellular Ca^2+^ cycling depends on the relationships between the various channels and pumps that are involved (Eisner et al., 2017). Phase 2 and 3 of the action potential correspond to the ST segment and T wave, respectively. These coincide with the rise and fall of intracellular calcium that governs cardiac myocyte contractility (Monasky et al., 2018). Much of the calcium enters the cell via L-type calcium channels, while an additional amount of calcium enters the cell via sodium-calcium exchange (NCX) channels. Calcium that enters the cell through both of these mechanisms triggers release of calcium from the sarco(endo)plasmic reticulum. Alterations in calcium handling could result in mechanical abnormalities, since calcium links the electrical and mechanical functions of the cell. An increase in a risk for arrhythmic events has been observed while patients with BrS were engaging in activities related to parasympathetic stimulation (Monasky et al., 2018), which results in an elevated ST segment, possibly through a reduction in I_Ca−L_ (Litovsky and Antzelevitch, 1990; Meregalli et al., 2005; Hoogendijk et al., 2011; Monasky et al., 2018). The reduced heart rate during parasympathetic stimulation results in a decrease in intracellular calcium amplitude (Hiranandani et al., 2006; Varian and Janssen, 2007). *CACNA1C* mutations could lead to a reduced intracellular concentration of calcium able to bind to troponin C of the myofilaments, thus disrupting excitation-contraction coupling (Monasky et al., 2018), the extent to which is still unclear. In fact, the induction of the BrS pattern has been associated with reduced contractility, particularly in the anterior free wall of the outflow tract, and reduced right ventricular ejection fraction (Pappone et al., 2019, 2020b). Therefore, further investigation of the role of calcium channel genes in BrS is warranted.

Antzelevitch et al. (2007) first described loss-of-function mutations in the LTCC genes *CACNB2b, CACNA2D1*, and *CACNA1C* in association with familial sudden cardiac death syndrome, the phenotype combining BrS and shorter-than-normal QT intervals. A role for *CACNA2D1* as a contributing factor in cardiac sudden death associated with a short QT interval has been described by significantly decreasing the cell surface protein expression of CaVα2δ (Bourdin et al., 2015). Importantly, in that study, the most significant reduction in CaVα2δ cell surface density was achieved by the combined effect of two genetic variants with little individual impact, highlighting the importance of polymorphisms. In fact, several other studies have highlighted the importance of common polymorphisms as genetic modulators of BrS (Lizotte et al., 2009), explaining the variable expression of the BrS phenotype (Wijeyeratne et al., 2020). Thus, in addition to rare mutations, also polymorphisms in calcium channel genes should be considered in future BrS research.

In their study, Di Mauro et al. (2020) state that *CACNA1C* mutations are the second most common cause of BrS. However, studies differ, likely due to differences in the gene panels used to screen patients, as well as the size and characteristics of the patient population. For example, in a recent report, variants in the *CACNA1C* gene were identified in about 7% of BrS patients who tested positive during genetic testing but who did not harbor variants in the *SCN5A* gene, making *CACNA1C* the fifth most popular gene screened after *SCN5A, AKAP9, SCN10A*, and *MYBPC3* (Pappone et al., 2020a). *AKAP9* encodes for A-kinase anchoring protein 9, a signaling protein that binds to the regulatory subunit of protein kinase A and has been implicated also as a genetic modifier of congenital long-QT syndrome type 1 (De Villiers et al., 2014). *SCN10A* encodes for the sodium voltage-gated channel alpha subunit 10. *MYBPC3* encodes for the myosin-associated protein cardiac myosin-binding protein C, which is involved in the regulation of force production and can be regulated by protein kinase A (Yang et al., 2001). Another study investigating the frequency of variants found in BrS patients also reported a higher frequency in *CACNA1C* compared to *SCN10A*, with variants in *CACNA1C* present in 2.6% of BrS patients overall, and 3.3% of BrS patients negative for variants in *SCN5A* (Di Resta et al., 2015). However, yet another study specifically looking at mutations in the genes *CACNA1C* and *CACNB2b*, encoding the α1- and β2b-subunits of the cardiac L-type calcium channel, respectively, found that 8.5% of the patients had mutations in at least one of these genes, although it is unclear how many harbored mutations in *CACNA1C* vs. how many harbored mutations in *CACNB2b* (Antzelevitch et al., 2007). Also, it was unclear if patients harbored mutations in other genes. However, regardless, it is clear by many studies that calcium channel variants have been found across various studies, by various authors, with various patient populations.

BrS is increasingly being recognized as an oligogenic disease (Monasky et al., 2020), with mutations in the *SCN5A* gene being more useful as a prognostic indicator, rather than a diagnostic one (Ciconte et al., 2020). To date, there is much that remains to be discovered about BrS genetics. Future studies need to identify and test new candidate genes. The genetics of BrS likely varies greatly from family to family, highlighting our need to move toward personalized medicine in BrS. Physiological studies such as the one by Di Mauro et al. (2020) are a good first step toward confirming the pathological effects of particular variants and treating patients with individual variants. However, much work remains before new pharmaceuticals can be developed, tested, and safely used in the clinic.

In conclusion, the study by Di Mauro et al. (2020) provides strong evidence of a possible gene-specific treatment in the future for BrS patients and is the first example of an LTCC-targeting therapeutic molecule that can correct I_Ca_ defects through modulation of channel density at the plasma membrane. Although preliminary, this is a promising step toward the development of pharmacological therapies to treat conductance abnormalities of the heart.

## Author Contributions

MM suggested the project and significantly reworked the article and provided guidance. CR wrote the first draft. EM and CP provided useful feedback. CP obtained financial support. All authors contributed to the article and approved the submitted version.

## Conflict of Interest

The authors declare that the research was conducted in the absence of any commercial or financial relationships that could be construed as a potential conflict of interest.
